# Pseudocholinesterase as a Biomarker for Untreated Wilson’s Disease

**DOI:** 10.3390/biom12121791

**Published:** 2022-11-30

**Authors:** Harald Hefter, Max Arslan, Theodor S. Kruschel, Max Novak, Dietmar Rosenthal, Sven G. Meuth, Philipp Albrecht, Christian J. Hartmann, Sara Samadzadeh

**Affiliations:** 1 Departments of Neurology, University of Düsseldorf, Moorenstrasse 5, 40225 Düsseldorf, Germany; 2 Departments of Anesthesiology, University of Düsseldorf, Moorenstrasse 5, 40225 Düsseldorf, Germany; 3 Experimental and Clinical Research Center, Charité–Universitätsmedizin Berlin, Corporate Member of Freie Universität Berlin and Humboldt-Universität zu Berlin, 10117 Berlin, Germany

**Keywords:** Wilson’s disease, cholinesterase, biomarker, heterozygotic gene carriers, diagnosis of Wilson’s disease

## Abstract

The aim of this study was to demonstrate that pseudocholinesterase (CHE) serum level is a useful diagnostic biomarker for untreated Wilson’s disease (WD). Between 2013 and 2019, about 75 patients were referred to the outpatient department of the University of Düsseldorf with suspected Wilson’s disease. In 31 patients with suspected Wilson’s disease (WD-SUS-group), WD was excluded by means of investigations other than analysis of blood and urine. A total of 27 parameters of blood and urine in these 31 patients were compared to those of 20 de novo patients with manifest WD (WD-DEF-group), which parameter showed the highest significance level of difference between the WD-DEF-group and the WD-SUS-group. Thereafter, receiver operating characteristics (ROC-curves) were analyzed to evaluate which parameter showed the largest area under the curve (AUC) to detect WD. Finally, a logistic regression analysis was performed to analyze which combination of parameters allowed the best classification of the 51 patients either into the WD-DEF-group or into the WD-SUS-group. CHE showed the highest significance level for a difference between the WD-DEF- and WD-SUS-group, had the highest AUC, and, in combination with ceruloplasmin, allowed 100% correct classification. Without CHE, no other combination of parameters reached this level of correct classification. After the initiation of treatment, which regularly results in an improvement in CHE, the high diagnostic accuracy of this biomarker was lost. Cholinesterase turns out to be an excellent biomarker for differentiation between untreated de novo patients with manifest WD and heterozygotic gene carriers.

## 1. Introduction

Wilson’s disease (WD) is an autosomal recessively inherited disorder of copper metabolism [1,2]. Copper is taken up from the gut, intravenously carried to the liver and actively transported into the hepatocytes via the human copper transporter (hCTR/CTR1) [3]. Intracellularly, antioxidant 1 copper chaperones (ATOX1) mediate copper to the P-adenotriphosphatase ATP7B in the trans-Golgi network, which facilitates the incorporation of copper into apo-ceruloplasmin to produce the copper transporting ceruloplasmin [4,5]. Ceruloplasmin intracellularly regulates iron metabolism and therewith the energy supply of the hepatocytes, delivers copper extracellularly to non-hepatic cells and mediates the excretion of copper from hepatocytes into bile [6,7].

In WD, the large ATP7B coding gene localized on chromosome 13 (13q14.3-q21.1) is mutated [8,9,10]. Hence, the synthesis of ceruloplasmin and the biliary excretion of copper are reduced, and the liver is overloaded with copper. The serum levels of unbound copper are elevated, resulting in tissue accumulation of toxic copper ions [11,12] and the impaired function of multiple organs, especially of the liver, kidneys and blood [12,13,14]. Due to the additional protection of the blood–brain barrier (BBB), an impairment of the central nervous system (CNS) usually occurs after hepatic manifestation. Thus, WD-patients with hepatic symptoms are diagnosed earlier than those with neuropsychiatric symptoms [15,16].

The diagnosis of WD may be difficult because the clinical manifestation of WD may begin with uncharacteristic symptoms such as fatigue and reduced motivation and motor skills. These symptoms, occurring during adolescence, may have a broad spectrum of organic and non-organic reasons. Therefore, sensitive algorithms to diagnose WD are very much appreciated. In 2012, a new diagnostic scoring system (Leipzig score (LS)) was introduced and published in guidelines for WD [17]. The complete LS is based on clinical findings, instrumental investigations and the analysis of various biochemical parameters. A total score of 4 or more indicates WD is highly likely, a score from 2 to 3 indicates WD is probable and a score <2 indicates WD is unlikely [17,18]. In case of an LS from 2 to 3, more investigations are required, which should be highly specific for WD (more information is presented in the discussion).

After the introduction of the LS in 2012, the number of patients that were referred to our center with suspected or “newly diagnosed Wilson’s disease” rapidly increased from 2013 on. However, in most of these “WD-patients”, the diagnosis of WD could not be confirmed. We, therefore, looked for a sensitive but easy-to-determine biomarker or a combination of biomarkers for WD, which can be used during a first screening visit to reduce the number of false positive WD diagnoses. Ceruloplasmin definitely is such a sensitive diagnostic marker [19,20], and 24 h urinary copper excretion has been used as the gold standard for diagnosing and monitoring therapy in WD over decades [14,15,20].

However, during the last years, pseudocholinesterase has turned out to be an excellent biomarker for liver cirrhosis [21,22]. It is highly sensitive and detects liver impairment in people exposed to pesticides [23], chronic [24] and acute [25] heart failure and is inversely correlated with the course of COVID-19 pneumonia severity and mortality [26]. These results suggest that serum levels of CHE may be helpful in differentiating between heterozygous WD gene carriers without hepatic involvement but with other neurological comorbidities and patients with definite WD who usually have subclinical or clinically overt hepatic involvement.

Compared to normal subjects, a variety of findings in the blood and urine of patients with WD usually show significant differences [20]. However, in heterozygous WD gene carriers, ceruloplasmin and therewith the serum levels of copper may be slightly reduced below the normal limit, whereas 24 h urinary copper excretion is normal. Therefore, we looked for a biomarker that is different in patients with manifest WD and patients with suspected but not confirmed WD as heterozygous gene carriers with neurological comorbidities. We compared a variety of biomarkers, such as serum copper, 24 h urinary copper excretion, ceruloplasmin, liver enzymes, including CHE between WD-patients being newly diagnosed and patients who were referred with suspected WD between 2013 and 2019 in whom WD was excluded by means of investigations other than the analysis of blood and urine.

This retrospective study was performed according to the declaration of Helsinki and approved by the local ethics committee of the University of Düsseldorf (Germany).

## 2. Methods

### 2.1. Patients with Definite WD (WD-DEF-Group)

In the outpatient department of the University hospital in Düsseldorf (Germany), a special ambulance for rare metabolic diseases was implemented in 1985. About 1 to 3 de novo WD-patients per year had presented in this institution since then. For the present study, 20 de novo WD-patients diagnosed in our department were recruited (WD-DEF-group). Demographical, treatment-related data and clinical, as well as laboratory findings, were collected (by TS Kruschel). Diagnosis of WD was based on typical biochemical findings, genetic testing, analysis of Kayser–Fleischer (KF) rings, cranial magnetic resonance imaging (cMRI scans), anterior segment optical coherence tomography (AS-OCT) [27] and acoustic radiation force impulse investigation (ARFI) [28,29]. An analysis of the biochemical parameters of blood and urine was performed for all 20 patients. Ten of these twenty WD-patients had a MELD score > 10.

### 2.2. Patients with Suspected WD (WD-SUS-Group)

Between 2013 and 2019 about 75 patients were referred to the special ambulance for rare metabolic diseases with suspected WD. A total of 75 charts were screened by M. Arslan (MA). In 14 of these patients, WD was confirmed. These 14 patients were included in the WD-DEF-group.

MA further detected 31 patients with probable WD according to the LS in whom WD was excluded by means of further investigations other than the 27 parameters of blood and urine used for therapy monitoring of WD (see Section 2.4 biochemical parameters). These 31 patients were included in the present study as the WD-SUS-group.

A total of 30 patients with suspected WD were excluded from the present study because: (i) they had refused to undergo further investigations or (ii) biochemical test parameters (used in the present study) were already used to exclude WD, such as 24 h copper excretion.

### 2.3. Clinical Neurological Findings in the WD-DEF- and WD-SUS-Group

For both patient groups (WD-DEF and WD-SUS), the main clinical findings were distributed to 10 different symptom categories: In the ASYMPT category, patients did not have hepatic or neurological symptoms. In the HEP category, patients had hepatic symptoms. Eight neurological categories were distinguished—N-TRE: patients with predominant tremor; N-PARK: patients with a juvenile parkinsonian syndrome; N-OTHMD: patients with other movement disorders such as generalized dystonia, chorea or ataxia; N-PAIN: patients with predominant pain syndromes; N-INFL: patients with inflammatory signs; N-FATIQUE: patients with a fatigue syndrome; N-DEGEN: patients with neurodegeneration; N-PSYH patients with depression (see Table 1).

### 2.4. Biochemical Parameters

In the outpatient department for rare metabolic diseases of the University hospital, patients with WD were monitored dependent on the severity of symptoms. More severely affected patients were seen every 3 months, and patients in a stable phase of the disease usually were seen once a year. During each visit, blood and urine samples were taken, and a list of 27 laboratory parameters was determined for routine therapy monitoring in WD. This list included: (1) parameters of copper metabolism (ceruloplasmin, serum copper, copper concentration in 24 h urine collected under medication, 24 h copper excretion in the urine); (2) liver enzymes (AST, ALT, GGT, CHE); (3) kidney function (serum level of creatinine (Crea)); (4) coagulation (counts of thrombocytes, thromboplastin time (PTT), Quick’s test). However, further parameters, such as albumin, leucocyte counts, or hemoglobin, were also determined at each visit.

These parameters were determined in both the untreated WD-DEF-group and the WD-SUS-group. We emphasize that these parameters were not used for classifying these patients into the WD-SUS-group (suspected but not confirmed WD). In the WD-DEF-group, these parameters, determined at the last visit before recruitment, were also included as data after WD-specific treatment.

### 2.5. Statistics

One-way ANOVA was used to detect significant (*p* < 0.05 after alpha adjustments because of multiple comparisons) differences of 27 parameters of blood and urine between the untreated WD-DEF-group and the WD-SUS-group. A further ANOVA was performed to find significant differences in the same parameters between the WD-DEF-group, after WD-specific treatment, and the WD-SUS-group.

For the combined data of the untreated WD-DEF- and WD-SUS-patients, receiver operating characteristic (ROC) curves were calculated for those 7 parameters showing the highest significant differences between the WD-DEF-group and the WD-SUS-group and for albumin and the hemoglobin. The area under the curve was determined for these parameters. A second ROC-curve analysis was performed for the combination of WD-DEF-patients, after WD-specific treatment, and WD-SUS-patients.

Finally, a logistic stepwise regression analysis (LSRA) was performed, which parameter or which combination of parameters yielded the highest probability, to classify untreated WD-DEF- and WD-SUS-patients correctly. A second LSRA was performed analyzing the probability of the correct classification of the treated WD-DEF-group and the WD-SUS-group.

ANOVA and LSRA were part of the commercially available SPSS statistics package (version 25: IBM Analytics, Armonk, NY, USA).

## 3. Results

### 3.1. Demographical Data and Spectrum of Clinical Symptoms in the WD-DEF-Group

The mean age at the manifestation of WD in the WD-DEF-group (*n* = 20) was 24.6 years (SD: 8.48). In 19 patients, WD had manifested well before the age of 35 years. Only seven (35%) patients in the WD-DEF-group were males.

In Table 1 (left part), the presenting clinical symptoms at manifestation are summarized. The most frequent presenting symptom in the WD-DEF-group was tremor (35%). A juvenile parkinsonian syndrome was observed in five patients (20%), whereas only one patient was totally asymptomatic in the WD-DEF-group. Compared to other centers with a large WD outpatient department, patients with clinically manifest liver dysfunction were underrepresented (20%).

### 3.2. Demographical Data and Spectrum of Clinical Cymptoms in the WD-SUS-Group

The mean age in the WD-SUS-group was 42.0 years (SD: 12.5). Ten patients were older than 50 years, and three were older than 60 years at the presentation in our clinic. Only 16 patients were within the 98% CI age range of the WD-DEF-group. Although about half of these patients presented with an atypical age for WD, we did not classify patients according to their age but according to additional clinical findings. A total of 13 (41.9%) patients in the WD-SUS-group were males.

In most of the 10 patients with atypical clinical findings for WD (grey area in Table 1 (right side)), diseases other than WD could be detected by scintigrams or lumbar puncture. In the 13 patients presenting symptoms similar to patients with WD, the performance of a DAT scan turned out to be very helpful in diagnosing idiopathic PD. Patients without symptoms underwent cranial MRI scanning, testing for KF rings and abdominal ultrasound investigation. Hepatitis was excluded in 30 out of 31 patients and confirmed only in 1 patient. In one patient, it was difficult to exclude WD during the first year of follow-up, but, finally, MSA was diagnosed due to the development of severe autonomic failure.

In summary, on the basis of clinical findings, MRI and DAT scanning, testing of KF rings, abdominal ultrasound investigation and the follow-up of other diseases, WD could be diagnosed in all 31 patients in the WD-SUS-group without the use of the 27 parameters of blood and urine.

### 3.3. Analysis of Blood and Urine in the WD-SUS-Group and in the WD-DEF-Group before Therapy

On their first visit to our institution, all patients underwent a detailed analysis of 24 blood parameters and 3 urinary parameters (for details, see Methods). These parameters were not used for the stratification of the patients into the WD-DEF- or WD-SUS-group.

One-way ANOVA was performed for all 27 parameters. Those seven parameters with the highest *p*-values, for a significant difference between the WD-SUS- and WD-DEF-group before WD-specific therapy, are presented in Table 2 (left side). Cholinesterase had the highest *p*-value (CHE: 4.58 × 10^−14^), followed by ceruloplasmin (Cerulo: 1.69 × 10^−7^), thrombocyte counts (Thromb: 1.80 × 10^−6^) and Quick’s test (Quick: 2.36 × 10^−5^).

**Figure 1:**
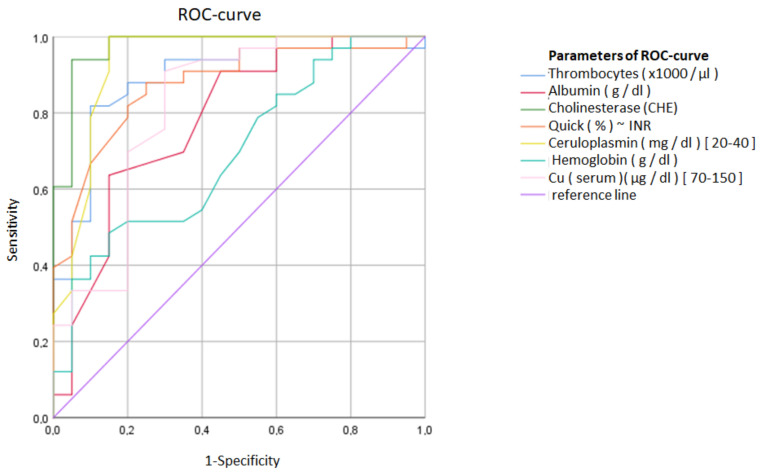
ROC-curves of the WD-DEF-group before therapy and the WD-SUS-group are presented for the following 7 parameters: counts of thrombocytes (thrombocytes), serum albumin (albumin), cholinesterase (CHE), Quick’s test (Quick), ceruloplasmin, hemoglobin and serum copper (Cu (serum)). The largest area under the curve (AUC) was found for CHE.

In the second step, the data of the WD-SUS-group and the data of the WD-DEF-group before therapy were lumped together, and it was tested which of these seven parameters and the copper concentration in 24 h urine (Cu (µg/L)) had the largest area under the ROC-curve analyzing sensitivity and specificity to predict the definite diagnosis of WD (see Figure 1). The largest area under the curve was found for cholinesterase (CHE: 0.97), followed by the 24 h urinary copper excretion (Cu (24 h): 0.96), the copper concentration in the 24 h urine (Cu (µg/L): 0.95) and ceruloplasmin (Cerulo: 0.93) (Table 2 (right side)).

### 3.4. Analysis of Blood and Urine in the WD-SUS-Group and in the WD-DEF-Group after Therapy

A second one-way ANOVA was performed comparing all 27 parameters of the WD-SUS-group and the WD-DEF-group after WD-specific therapy (Table 2 (right side)). The parameter with the highest *p*-value was CHE (8.12 × 10^−8^), followed by serum copper (2.49 × 10^−7^), ceruloplasmin (3.98 × 10^−7^) and the 24 h urinary copper excretion (Cu (24 h): 1.19 × 10^−5^).

When the parameters of the WD-SUS-group and the WD-DEF-group after WD-specific therapy were combined and tested, after which the parameter had the largest area under the ROC-curve, a different order of parameters was found (see Table 2). The largest area was found for serum copper (0.96), CHE (0.93), ceruloplasmin (0.91) and thrombocytes (0.89).

### 3.5. Probability for Correct Classification of WD-DEF- and WD-SUS-Patients

**Figure 2:**
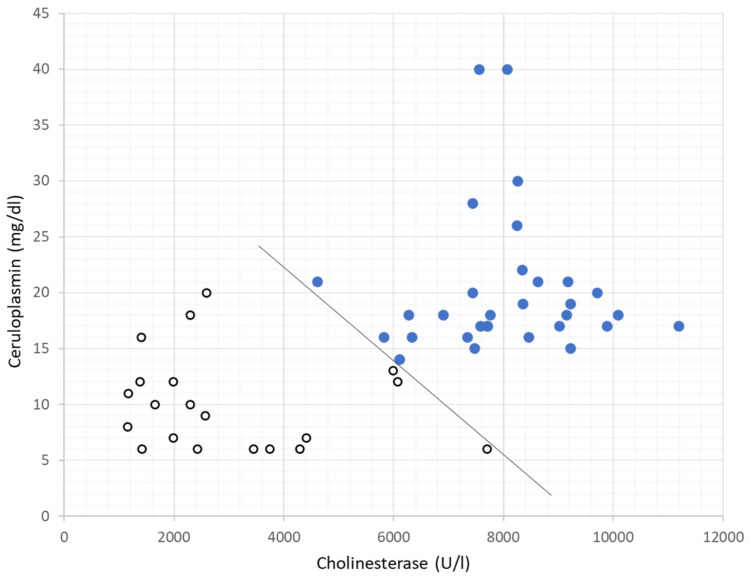
Ceruloplasmin (mg/dL) (ordinate) was plotted against cholinesterase (U/L) (absc issa) for the WD-DEF-group (open circles) and the WD-SUS-group (full circles). A linear combination of CHE and ceruloplasmin allowed the separation of the WD-DEF- and WD-SUS-group completely.

Logistic stepwise regression analysis (LRA) was performed by which the combination of parameters before WD-specific therapy could be used to yield a high probability of the correct classification of WD-DEF- and WD-SUS-patients. Among all single parameters, the cholinesterase yielded the best probability (92.5%) in classifying WD-DEF- and WD-SUS-patients correctly. By means of a linear combination of CHE and ceruloplasmin, 100% of all 51 cases were correctly classified. This is demonstrated in Figure 2.

When CHE was removed from the list of parameters for LRA and a second LRA was performed, the best probability to separate WD-DEF- and WD-SUS-patients was found with a combination of ceruloplasmin, thrombocytes and Quick’s test of 94.4%.

When a third LRA was performed, including CHE but using the parameters of the WD-DEF-group after WD-specific therapy, the best single parameter for correct classification was serum copper (88.46%). The linear combination of serum copper and CHE yielded a probability to classify WD-DEF- and WD-SUS-patients correctly of 98.1%. No other combination of parameters yielded a higher probability.

When CHE was removed from the list of parameters for LRA and a fourth LRA was performed, the linear combination of serum copper and thrombocytes yielded the best probability of 90.38%.

In summary, when data of the WD-DEF-group after WD-specific therapy were used, no combination of parameters could be detected, which allowed the complete separation of WD-DEF- and WD-SUS-patients.

## 4. Discussion

### 4.1. The Difficulty of Diagnosing WD

The spectrum of clinical symptoms and the spectrum of possible differential diagnoses is broad in Wilson’s disease [12,14,15,30]. The age of the onset of symptoms is a very helpful parameter in the differentiation between patients with WD, heterozygotic gene carriers or normal subjects. Neurological WD manifests in juvenile patients or young adults [12]. The mean age at the appearance of neurological symptoms is 20.2 (SD 10.8) years [31,32], and a delay between the onset of symptoms and a diagnosis of WD from 1 to 2 years has been reported [13]. The mean age at diagnosis was 24.6 years in the present study; therefore, this is in full agreement with other series of WD-patients with predominantly neurological manifestations [12,13,33]. The manifestation of WD beyond the age of 50 has been reported but is extremely rare [34].

As a general aspect, it should be kept in mind that WD is a rare disease. The manifestation of Parkinson’s disease (PD), with a prevalence of 0.1% at an age of 50 [35], or essential tremor (ET), with an overall prevalence of 0.9% [36], is much more likely than WD when patients experience the development of tremor. Therefore, patients with suspected WD should be seen by a movement disorder specialist.

Nevertheless, without biomarkers in blood or urine, the differentiation between patients with WD and heterozygotic gene carriers or normal subjects may be difficult. In the present study, we managed to do this by means of expensive additional investigations, such as DAT scans to detect pre-synaptic dopaminergic deficiency, MRI scans to detect WD-typical CNS lesions, slit-lamp and OCT investigations [27] to detect KF rings, lumbar puncture to exclude an inflammatory cause of symptoms and modern abdominal ultrasound investigation (ARFI) to detect liver cirrhosis [28,29].

Even more sensitive and more WD-specific tests have been developed recently. Whole-exome sequencing provides detailed information on the underlying genotype [37], the ultrafiltrable and exchangeable copper can be determined [38] and used as a new tool in the diagnosis of WD [39], the ATP7B protein can be quantified in dried blood spots [40], the incorporation of radioactive copper into ceruloplasmin can be tested [41] and the retention of radioactive copper in the liver can be analyzed by PET [42]. In general, these tests have an excellent sensitivity and specificity. ^64^Cu-PET differentiates highly significantly between patients with manifest WD, heterozygous gene carriers and healthy controls [42]. The ^64^Cu incorporation test has a sensitivity of 98.6% and a specificity of 100% [41]. However, even for these expensive and sophisticated tests, pitfalls may exist [43]. Alzheimer’s patients also present with an abnormal exchangeable copper component [44], and patients with two mutations on one allele do exist [43,45]. Therefore, Czlonkowska et al. strongly recommend “that the diagnosis of WD must always be confirmed by clinical, laboratory and genetic compatibility” [45].

Compared to the performance of such additional investigations, the standard analysis of blood and urine is much cheaper. The question is whether a differentiation between patients with definite WD and patients with suspected but not confirmed WD, including gene carriers, can be performed equally well only by means of biomarkers in blood and/or urine.

### 4.2. Classification by Means of Biomarkers

In a recent detailed Cochrane review analyzing the applicability of ceruloplasmin, hepatic copper content and 24 h urinary copper excretion as biomarkers for WD, it was emphasized that the sensitivity and specificity of a biomarker are critically dependent on the cut-off limits for the normal range [20]. Such evaluations of limits are necessary before a scoring system, as the Leipzig score is widely applied to avoid false positive results. This is indeed a very important aspect because the re-evaluation of the diagnosis after the initiation of specific treatment may become even more complex than establishing the diagnosis (see discussion below).

The serum level of ceruloplasmin can be altered by hormonal treatment and inflammation. The sensitivity of ceruloplasmin lies between 77.1 and 99%, with specificity between 55.9 and 82.8% when a cut-off of 20 mg/dL is used. A cut-off limit of 10 mg/dL seems to reduce sensitivity from 65 to 78.9% but increases the specificity from 96.6 to 100% [20]. The 24 h urinary copper cut-off is critically dependent on the duration of the withdrawal of medication and oral copper uptake.

We, therefore, prefer to use combinations of parameters rather than score whether a single parameter lies in or outside the normal range. In heterozygous gene carriers, some parameters may lie outside the normal range, such as ceruloplasmin or serum copper, but others may lie inside the normal range, such as 24 h urinary copper excretion.

### 4.3. Classification Using Pseudocholinesterase (CHE)

In the present study, pseudocholinesterase turned out to be the best parameter to differentiate between patients with definite WD and patients with suspected WD, who were probably heterozygotic gene carriers and/or suffered from other mainly neurological disease entities. More than 92% of WD-DEF- and WD-SUS-patients were classified correctly by means of CHE, although patients with hepatic symptoms were underrepresented in the WD-DEF-group. This is no surprise because CHE is a sensitive biomarker for liver cirrhosis [21,22] and patients with manifest WD have a subclinical or clinically overt hepatic involvement. CHE is used to detect subclinical liver dysfunction in people who were exposed to pesticides [23], is used as a sensitive marker in patients with chronic [24] and acute [25] heart failure and as a prognostic factor in COVID-19 infection [26]. In most WD-patients, liver function is impaired because of the toxic influence of copper on the hepatocytes. Therefore, a mild reduction of CHE has to be expected early in the course of WD.

### 4.4. 100% Correct Classification by Means of CHE and Ceruloplasmin

The ATP7B gene is necessary to produce intact ceruloplasmin. In WD, the ATP7B gene is mutated, and low ceruloplasmin levels are typical for WD [13]. Ceruloplasmin differentiates well between WD-DEF- and WD-SUS-patients but not as well as CHE (see results). However, both parameters in combination allowed 100% correct differentiation between WD-DEF- and WD-SUS-patients (Figure 2). Because the altered serum levels of CHE and ceruloplasmin are characteristic parameters for WD and result from different aspects of the pathogenesis of WD, the combination of these two parameters adds information for the separation of WD-patients and heterozygotic gene carriers and is more sensitive than each of these two parameters. CHE is not a specific biomarker of WD. As mentioned in the introduction, CHE is a sensitive biomarker of liver cirrhosis [21,22]. Thus, CHE indicates hepatic involvement in WD-patients even when manifest hepatic symptoms are missing.

### 4.5. Cholinesterase Deficiency Syndrome as a Pitfall for the Use of CHE as a Biomarker for WD

A possible pitfall for the use of cholinesterase as a biomarker for WD is genetically determined cholinesterase deficiency syndrome (CHEDS; [46]). These patients have reduced CHE but no other hint of liver dysfunction. We came across this problem in a family with three children; one male child had WD only, one female child had CHEDS only and one male child had both WD and CHEDs. In both male children, CHE was low in the beginning; in the elder brother, CHE recovered; in the other patient, CHE remained low. Therefore, WD patients with a persistent low level of CHE, despite sufficiently high copper chelating therapy, should be tested for the presence of CHEDS because this has high clinical relevance for the patient (for details see [47]). The prevalence of CHEDS is about 3–4% in the European population [46]. Therefore, the combination of CHEDS and WD is extremely rare [48].

### 4.6. Probative Treatment does Not Improve Classification

It has been recommended that copper chelating agents in unclear cases of suspected WD are applied. As demonstrated above, the clear separation between patients in the WD-DEF- and WD-SUS-group was lost with the ongoing treatment of the WD-DEF-group. No combination of parameters was found to allow the correct classification of WD-SUS-patients and WD-DEF-patients after WD-specific therapy. CHE improves with WD-specific treatment (Table 2 (right side)), with the implication that the high precision for correct classification is reduced. Serum copper separates WD-SUS-patients from treated WD-patients better than or at least as good as CHE because copper chelating therapy decreases serum copper, thereby improving its ability to separate WD-DEF-patients from WD-SUS-patients. After therapy, no combination could be detected, which allowed 100% correct classification or separation of WD-DEF- and WD-SUS-patients.

### 4.7. Clinical Recommendation

For the diagnosis and therapy control of WD, we recommend determining CHE and ceruloplasmin early before expensive investigations are ordered. In WD, CHE is low, as is ceruloplasmin. We do not recommend using probative treatment to improve copper excretion. The initial dose of copper chelating agents has to be low. The initial dose may be too low to increase 24 h urinary copper sufficiently. We, therefore, think that it is better to wait a further 3 months and then repeat the measurement of various biomarkers again. In the meantime, genetic testing, slit-lamp investigations to detect KF rings, MRI scans and even more recent and sophisticated tests can be performed, which are also very helpful in the differential diagnosis of neurological WD.

### 4.8. Strengths and Limitations of the Study

The strength of this study is that the hypothesis was confirmed; CHE can be used as a cheap, sensitive biomarker to differentiate between patients with untreated manifest WD and patients with an LS score between 1 and 3, in whom WD could not be confirmed. However, our study was retrospective, and the number of de novo WD-patients was rather small. Furthermore, only 4 patients in the WD-SUS-group had hepatic symptoms, whereas 10 in the WD-DEF-group had a MELD score > 10, although patients with clinical hepatic symptoms were underrepresented in the WD-DEF-group. This might have caused a bias in the results towards an overestimation of the discrimination ability of CHE. Therefore, a prospective, multicenter study should be performed to confirm the present results. Furthermore, the genetic information in the WD-SUS-group was poor. We, therefore, recommend that this multicenter study should be combined with modern panel mutation analysis and at least one modern, highly specific and sensitive instrumental test, following the recommendation by Czlonkowska et al. that the diagnosis of WD is founded on a broad base of clinical, laboratory and genetic data [45].

## Figures and Tables

**Table 1:** Main presenting symptoms in the WD-DEF-group (left part) and in the WD-SUS-group (right part).

		WD-DEF-Group		WD-SUS-Group	
Clinical subgroup	*n*=	Main symptom	*n*=	Main symptom	Final diagnosis
ASYMPT	1	n.a.	8	n.a.	Gene carrier
HEP	4	2 patients with reduced daily activities 1 patient with intellectual decline 1 patient with acute hepatic failure	1	1 patient with reduced daily activities	Hepatitis
N-TRE	7	Tremor of extremities and trunk	6	3 patients with symmetric tremor of hands and head 3 patients with asymmetric hand tremor	Essential tremor Idiopathic PD
N-PARK	5	Symmetric bradykinesia	2	Reduced arm swing	Idiopathic PD
N-OTHMD	3	Cerebellar ataxia, Chorea, generalized dystonia	4	Psychogenic movement disorder	Confirmed
N-PAIN	0		3	Pain in muscles and joints	Rheumatism, joint degeneration, Complex regional pain syndrome, CRPS
N-INFL	0		3	Paresthesia, spasticity	Neuroinflammatory disease (MS, NMO)
N-FATIQUE	0		2	Severe tiredness	MS, CFS
N-DEGEN	0		1	Loss of balance	MAS
N-PSYCH	0		1	Fatigue	Depression

The clinical subgroups or symptom categories are explained in more detail in Methods. PD—Parkinson’s disease; MS—multiple sclerosis; NMO—neuromyelitis optica spectrum disease; CFS—chronic fatigue syndrome; and MAS—multi system atrophy. The symptoms of the 10 patients in the grey area of Table 1 are less typical for WD than tremor, parkinsonism or other movement disorders.

**Table 2:** Comparison of parameters of blood and urine in the WD-DEF- and WD-SUS-group.

Area under ROC-Curve	Rank	Parameter	WD-DEF MV/SD before Therapy	*p*-Value	WD-SUS MV/SD	*p*-Value	WD-DEF MV/SD after Therapy	Rank	Area under ROC-Curve
0.97	1	CHE	2999/1801	4.58 × 10^−14^	8035/1388	8.12 × 10^−8^	5048/1634	1	0.93
0.93	2	Cerulo	10.1/4.1	1.69 × 10^−7^	20.2/6.4	3.98 × 10^−7^	9.7/5.1	3	0.91
0.89	3	Thromb	139/57	1.80 × 10^−6^	249/72	1.19 × 10^−5^	154/47	5	0.89
0.88	4	Quick	68.3/22.1	2.36 × 10^−5^	93.5/15.4	2.89 × 10^−3^	79.3/17.0	7	0.86
0.82	5	Cu (serum)	51.6/27.2	1.96 × 10^−4^	93.0/36.8	2.49 × 10^−7^	35.1/22.1	2	0.96
0.96	6	Cu (24 h)	966/1292	4.42 × 10^−4^	31.0/42.5	1.19 × 10^−5^	160.1/138.1	4	0.88
0.88	7	GPT	91.0/95.1	1.17 × 10^−3^	24.0/16.1	3.38 × 10^−2^	47.7/47.1	>7	0.65
0.95	>7	Cu (mg/L)	519/753	1.47 × 10^−3^	24.5/44.8	6.76 × 10^−4^	113.3/121.2	6	0.86

ROC-curve—receiver operating characteristics curve; MV—mean value; SD—standard deviation. The values of the WD-SUS-group (in the middle) were compared with those of the WD-DEF-group before therapy (left side) and after WD-specific therapy (right side). *p*-values are presented in the special exponential format. Parameters were ranked according to the *p*-values for comparison of the parameters before therapy (left part). After therapy, the ranking of the parameters’ changes (right part). The area under the ROC-curve for the parameters mentioned in column two are presented in the first column for the data before therapy and in the last column after therapy.

## Data Availability

Data available on request due to restrictions, e.g., privacy or ethics. The data presented in this study are available on request from the corresponding author.
